# Multiscale Interfacial Structure and Organization of sII Gas Hydrate Interfaces Using Molecular Dynamics

**DOI:** 10.3390/nano15060464

**Published:** 2025-03-19

**Authors:** Samuel Mathews, Phillip Servio, Alejandro Rey

**Affiliations:** Department of Chemical Engineering, McGill University, Montreal, QC H3A 0C5, Canada; samuel.mathews@mail.mcgill.ca (S.M.); phillip.servio@mcgill.ca (P.S.)

**Keywords:** multiscale, nonlinear, gas hydrate, clathrate, molecular, interface, dipole

## Abstract

Gas hydrate systems display complex structural arrangements in their bulk and interfacial configurations. Controlling nucleation and growth in the context of potential applications requires a characterization of these structures such that they can be manipulated at the atomic and molecular scale to fine tune macroscale applications. This work uses molecular dynamics to show the different methods of identifying interface location and thickness, the drawbacks of certain methods, and proposes improved methodology to overcome sampling issues. We characterize the interfacial position and thickness using structure and dipole-based methods at different conditions for water/sII natural gas hydrate mixtures. We find that phases with similar densities are particularly sensitive to the regression technique employed and may not resolve the thickness of the complex pre-melting layer adequately, while the dipole moments may provide better resolution. The dipole shows the complex natural of the small and compressed layer that presents on the hydrate surface. These results show that the interface is thin but dynamic and careful characterization required analysis of multiple molecular phenomena.

## 1. Introduction

At low temperatures, high pressures, and in the presence of gaseous molecules, water molecules can come together and assemble to form gas hydrates. The resulting water backbone retains its structure through hydrogen bonding, and the guest molecules provide further stability through Van der Waals forces. Without the guest molecules, crystalline ice would form instead. These clathrates are generally found in petroleum transportation and extraction, where flow assurance problems and related safety concerns are their most critical consequence [1]. Gas hydrates are also studied for methane extraction, flue gas capture, desalination, natural gas capture, hydrogen storage, carbon capture and storage, and due to their prevalence planetary ices [2,3,4,5,6,7]. The formation and dissociation of hydrates are critical in engineering applications of these technologies.

Guest identity, pressure, and temperature are the three main considerations controlling gas hydrate formation, affecting the nucleation rate, growth rate, formation conditions, and crystalline structure selectivity [8,9,10,11,12,13]. These structures crystallize into three main forms: structure I (sI), structure II (sII), and structure H (sH). All three are composed of combinations of various polyhedra, creating cages around the guest molecules of various sizes. Small gaseous molecules like carbon dioxide, ethane, methane, and hydrogen sulfide typically form sI, with a unit cell made up of 46 water molecules organized into two smaller pentagonal dodecahedron ($5^{12}$) cages and six larger tetrakaidecahedron ($5^{12}6^{2}$) cages. Larger gas molecules like propane and isobutane, and certain gas mixtures like natural gas, can form sII, with a unit cell made up of 136 water molecules organized into 16 smaller $5^{12}$ cages and eight larger hexakaidecahedron ($5^{12}6^{4}$) cages. Other gas mixtures, mainly composed of larger gases like neohexene can form sH, with a unit cell made up of 36 water molecules organized into smaller $5^{12}$ cages, two irregular dodecahedron ($4^{3}5^{6}6^{3}$) cages, and one large icosahdron ($5^{12}6^{8}$) cage. The unit cells of sI and sII are cubic, while sH has a hexagonal unit cell [1,14].

The experimental determination of gas hydrate structures and behaviors took considerable time since they can form in nonstoichiometric proportions, leading to variations due to different guest molecule populations [1,15,16]. The most common structure in flow assurance is the sII structure as natural gas is composed mainly of methane and ethane, with the methane filling the small $5^{12}$ cages and the ethane filling the large $5^{12}6^{2}$ cages [17]. Careful considerations of the formation behaviors and structures of sII particularly, but also sI and sH, are necessary because the structures can undergo solid phase transitions when conditions change. For example, certain predictions show that only 0.3 mole percentage of ethane is required to cause the sII structure to become sI. This potential for solid transition creates nonlinear parameter spaces that envelop the formation conditions, guest identity, as well as solid characteristics, surface and bulk stresses, and molecular interactions. Therefore, many subsea hydrate deposits could be sII instead of sI, and their actual properties may deviate from initial predictions based on incorrect assumptions regarding their structure [18].

Gas hydrates may form as films at interfaces, multiple crystals, or single crystals, following various pathways. These multistep pathways proceed at different timescales and various structures may form and deform during initial nucleation and during the sustained nucleation events, creating complex nonlinear behaviors in all measurables while studying these phenomena [19]. Usually, critical nuclei will initially form at the gas/water interface and continue along this surface [20]. This initial growth happens at the atomic scale and is difficult to observe in a laboratory setting [21]. As it continues, the structure displays dendritic growth, shell formation, and various multiscale and multistep dynamics [21,22,23,24]. As surface energy minimization and guest diffusion compete in the nucleation processes, dendrites, faceted crystallites, and porous media form. Characterizing these mesoscale (100 nanometers to 100 micrometer) structures requires information about the free energy landscape and surface forces coming from the molecular scale (less than 10 nanometers) [25]. This multiscale relationship between different characterization methods highlights the necessity for accurate information regarding the molecular behaviors involved [26,27]. This necessity is a consequence of treating nonlinear dielectric responses in hydrate films, dealing with complex interfacial water systems, considering the methane disorder in hydrate heat transport, correctly treating abrupt changes in density, potential energy, and molecule dipole across the interface, and dealing with the impact of pressure loads across scales, from bonds to lattices to continuous crystals [28,29,30,31].

Despite this need, thorough characterization of the molecular phenomena in phase transitions, hydrogen bonding systems, the design of surface-controlled materials like adhesives, and nucleation remained limited and incomplete [27,32,33,34,35,36]. The size and speed of systems at this scale restrict experimental methods, as does their anisotropy when stressed in certain directions. In protein hydration, for example, traditional methods of describing the structure are not suitable because translational and rotational symmetry is not preserved [37]. While simplified mesoscale laws may be attractive due to the system size they can treat, these require continuum laws as boundary conditions. In turn, the continuum laws need to capture main trends in molecular bonding and attachments that can only be elucidated with molecular dynamics (MD) and subsequently need to be fit against MD simulations to deal with the configurational aspects of molecules forming crystal structures [25].

The location and thickness of the interface is of particular interest in these problems. Various models rely on accurate measures of the position and thickness since they fundamentally affect physical, chemical, mechanical, and electrical behaviors. Thickness influences electron transport that is critical in semiconductor and nanomaterial design [38,39]. In MD and density functional theory (DFT), precise interface localization precludes accurate surface energy, reaction rates, and interface diffusion coefficient calculations [40]. At a larger scale, predicting the phase change dynamics or curvature effects in sharp interface models like the Stefan problem in solidification or the Young-Laplace equation for capillarity depend on knowing the interface position [41,42,43]. In chemical reactions, the interfacial geometry controls heat and mass transfer, dictating product distribution and reaction efficiency in catalytic and multiphase reaction systems [44,45]. At a macroscopic scale, when studying fluid mechanics and large-scale environmental problems, accurate measures of interface position and thickness form key parts of solving coupled equations governing fracture mechanics and multiphase flows, like oil-water migration or wave propagation [46,47]. In these multiscale domains, poor measures of the interface thickness and its location may lead to modeling errors that lead to inaccurate predictions and suboptimal designs.

Therefore, the use of MD techniques to characterize gas hydrates and their nucleation and growth gives researchers the opportunity to glimpse at the molecular behaviors while simultaneously allowing the treatment of a large enough system to draw conclusions regarding macroscale behaviors. Additionally, MD provides an avenue for treating complicated, nonlinear, and multiscale behaviors of gas hydrate nucleation and growth, and studying the surface stresses and molecular organization at the interfaces [29,48,49]. The main purpose of this computational study is to show the different methods of measuring interfacial position and thickness, explain the often-unstated drawbacks of using certain methods, propose novel techniques to acquire better estimates of these quantities, and reveal critical structures and behaviors at play in realistic engineering systems. This work will use MD to treat the nonlinear multiscale phenomena in gas hydrates. Additionally, studying the surface structure and molecular organization provides information for the crystal growth and nucleation of other polycrystalline systems, and how certain layers may ease transformations from solid to liquid and vice versa [50,51,52,53,54].

The present work is organized in the following way. The general computational techniques and details are described in the methodology. Descriptions of MD and numerical techniques used to calculate the various surface properties follows. Thereafter, results are presented and validated using experimental and theoretical data for discussing and validating the results. We calculate and analyze the interfacial thickness and the molecular ordering and scrutinize the different techniques for characterizing these in an effort to propose a more effective technique. Finally, the conclusions are presented, summarizing the results and discussing the implications of the findings.

## 2. Materials and Methods

**Figure 1:**
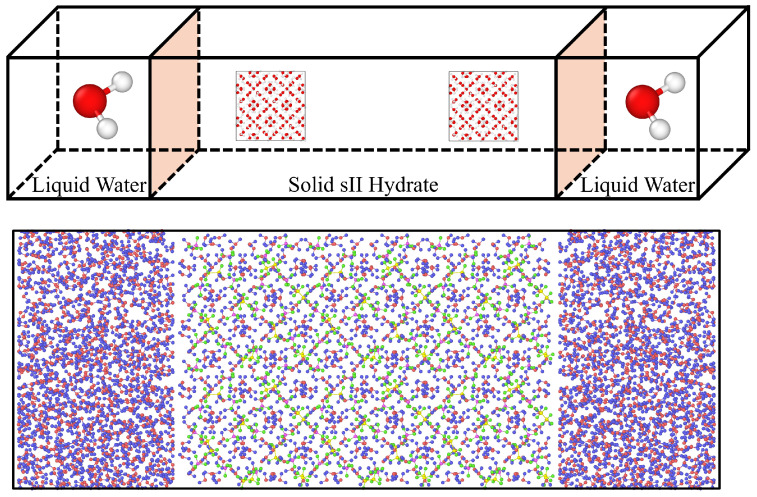
A schematic representation of the initial system configuration aligned with an initial molecular snapshot of the system trajectory.

We use the Large-scale Atomic/Molecular Massively Parallel Simulator (LAMMPS, stable release 2 August 2023) implementation of MD to explore the interfacial structures and properties of gas hydrate systems [55]. We follow the computational methods required in surface science to study these by confining the solid phase between two liquid water phases as shown in Figure 1 [56,57]. These methods have successfully characterized a variety of hydrate interfacial systems with accuracy and efficiency [23,29,49,58].

Ice and water structures possess certain quantum phenomena that cannot be capture by classical MD. For example, MD nuclei as classical particles, neglects the zero point energy, does not treat different isotopes apart from their masses, and cannot deal with quantum delocalization of protons. In the second coordination shell of water there is some increased structuring elucidated by quantum simulations, and the hydrogen bond structure is slightly affected, especially at low temperatures. However, even with the use of novel simulation methods and powerful computers, including the extensive use of graphical processing units, the computational cost of quantum simulations still limits the system size to hundreds of atoms, scratching the surface of the required sizes to simulate aqueous interfaces [59]. Therefore, we find classical MD to be the most suitable method for the study of the hydrate interfaces when appropriate steps are taken to ensure thermal equilibrium and adequate system size.

The system is composed of a solid phase of a methane/ethane gas hydrate, where the sII structure is fully occupied; the smaller cages are filled with methane gas and the larger cages are filled with ethane gas. The assumption of fully occupied ensures that the system is stable at the given conditions. Without full occupation, the system would decompose after a time and the results would not be representative of the system at the given conditions [60]. The hydrate is surrounded on both sides by boxes of pure liquid water and periodic boundary conditions throughout. This configuration improves property sampling by providing two clear interfaces in the system. To adequately sample the properties at different conditions, the system is simulated at different temperature and pressure combinations, ranging from 1.5 to 7.6 megapascals and 273.15 to 290.15 Kelvin. This narrow band of conditions is required to ensure that the simulations replicate realistic sII formation conditions and do not cross into sI conditions [18].

To begin, the simulation box has the dimensions of 40×100×40 Å in the *x*, *y*, and *z* directions. This corresponds to a hydrate phase of 12 unit cells of sII, with the lattice parameter of 17.31 Å. The coordinates of the clathrate are obtained from high resolution X-ray diffraction experiments to satisfy the necessary Bernal-Folder ice rules, possess a net dipole of zero, and provide the lowest potential energy configuration [61,62]. The liquid water boxes each contain 977 water molecules such that the density of water can be set to 1.0 $g\cdot {\mathrm{cm}}^{-3}$ at the initial conditions. As the simulation progresses, the simulation box will be allowed to expand and contract to accommodate the temperature and pressure changes. Figure 1 shows a representative image of the initial configuration of the system. Note that the buffer between the edges of the simulation box and the phases exists to permit molecular movement when the thermostat is initially applied.

The gas molecules are randomly oriented but centered in their respective cages. Water molecules forming the liquid water phase are placed randomly in the space they occupy. The water molecules constituting the hydrate phase are placed according to the aforementioned coordinates. The random placement of water molecules and the random orientation of guest molecules avoids imposing order while providing appropriate initial configurations for LAMMPS by minimizing short range repulsive interactions. This pre-processing step is performed with PACKMOL (v20.15.3) and reduces the computational cost of the simulation [63,64].

The cross-platform program Moltemplate (v2.22.2) was employed to accelerate the assignment of force field values and associated parameters, allowing for custom and complex manipulations of atom bonds, angles, and other quantities [65]. Methane and ethane molecules were modeled by the Optimized Potentials for Liquid Simulations—All Atom (OPLSAA) [66,67]. The improved four-site water model TIP4P/Ew was used to model the water molecules [68]. This version of the TIP4P parameters was chosen because its parameters have been adjusted for use with a long-range Coulombic solver and performs well in the vicinity of interfaces and for liquid and solid water [22,23,29,69]. The force field parameters are displayed in Table 1.

The Lorentz-Berthelot mixing rules applied to inter-molecular interactions and a cutoff of 12 Å was used for both the Lennard-Jones (LJ) and Coulombic interactions:

(1) $$E=4\epsilon \left[{\left(\frac{\sigma }{r}\right)}^{12}-{\left(\frac{\sigma }{r}\right)}^{6}\right]\;r<r_{c}$$

(2) $$E=\frac{Cq_{i}q_{j}}{\epsilon r}\;r<r_{c}$$

(3) $$\epsilon_{ij}=\sqrt{\epsilon_{ii}\epsilon_{jj}}$$

(4) $$\sigma_{ij}=\frac{\sigma_{ii}+\sigma_{jj}}{2}$$

A force error accuracy of $1\times 10^{-5}$ ensured accurate treatment of long-range interactions with the particle-particle particle-mesh solver of Hockney and Eastwood [70,71]. The Shake algorithm constrained the angles and bound lengths of water molecules in an effort to alleviate computational cost, and other angles and bonds were treated with the harmonic style. Postprocessing steps involving parsing, handling, and processing trajectories output from LAMMPS were performed using the Python (version 3.11.5) library MDAnalysis (version 2.8.0) [72,73,74].

We employ the Nosé-Hoover thermostat and barostat to control the temperature and pressure respectively and use appropriate damping values to reduce oscillations in these two quantities. Furthermore, we employ the isothermal-isobaric-iso-interface area (NP_N_AT) ensemble that imposes a constant normal pressure and constant cross-sectional area during the secondary part of equilibrium and data collection. This ensemble is helpful in equilibrating systems of this type, and overcomes issues with applying isotropic pressures to two-phase simulations [75,76]. We use the Verlet algorithm to integrate the non-Hamiltonian equations of motion with a timestep of 2 femtoseconds. The simulations run 100 nanoseconds to achieve equilibration, and then an additional ten nanoseconds over which data is collected in the latter half.

## 3. Results

### 3.1. Equilibration

**Figure 2:**
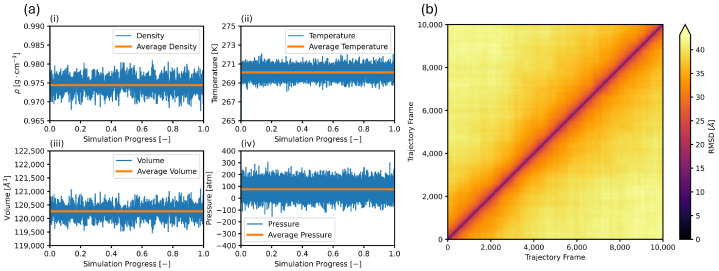
Different measures of assessing equilibrium. (**a**) Shows key time series data, sampling (**i**) density, (**ii**) temperature, (**iii**) volume, and (**iv**) pressure with time. (**b**) Show the all-to-all root mean square deviation (RMSD) of the simulation trajectory over ten nanoseconds. Plots are for 7.6 megapascals and 270.15 Kelvin. Samples are taken every two picoseconds.

The first step to MD studies should constitute an assessment of the system’s behavior nearing equilibrium, as this is often a condition of adequate sampling. Two strategies exist for assessing the behavior of the system nearing equilibrium: analyzing the time series of key parameters to assess that they fluctuate about a mean value and using distance measures to qualitatively evaluate configurational space sampling [77]. Executing the first strategy is centered around checking that various scalar values fluctuate around a mean value and show no appreciable drift. Common measures include temperature, pressure, density, and/or total energy [49,78,79,80]. The first panel of Figure 2 shows the density, temperature, volume, and pressure of the system over the final 10 nanoseconds of simulation time. The system density fluctuates between 0.970 $g\cdot {\mathrm{cm}}^{-3}$ and 0.980 $g\cdot {\mathrm{cm}}^{-3}$, a narrow range relative to the overall density of the system and always around the average density. The temperature and volume also fluctuate over small ranges around the mean values for the simulation. The pressure fluctuates over a wider range but still around the expected mean value for the simulation. Such fluctuations are expected thanks to the computational functioning of the barostat and are minimized by adequate selection of dampening and drag terms. The fluctuations of the scalar values above and below a mean value are important in displaying that a simulation system is sampling the parameter space thoroughly, visiting new states and previously visited states. The lack of drift with time of these quantities further solidifies the conclusion that the simulation is close to equilibrium.

Executing the second strategy involves calculating the all-to-all root mean square deviation (RMSD) of the simulation trajectory over sampling timeframe. In this case, the RMSD is the square root of the average of the square difference of all atomic positions during the interval. One can assess if the system undergoes large configuration changes during the simulation, as well as if the configuration drifts with time in a correlated manner using the comparison of all trajectory frames to each other (all-to-all). The second panel of Figure 2 shows the RMSD of the simulation trajectory over the final 10 nanoseconds of simulation time. The diagonal always shows 0 RMSD because the difference of every configuration with itself is null. Darker regions would indicate pockets of structures with some similarity. Off diagonal RMSD minimums display that the system samples previously visited states, a condition of proper sampling [77]. Figure 2 confirms that the system is at equilibrium, and all conditions show the same characteristics. Therefore, subsequent analysis can continue.

### 3.2. Interfacial Structure

**Figure 3:**
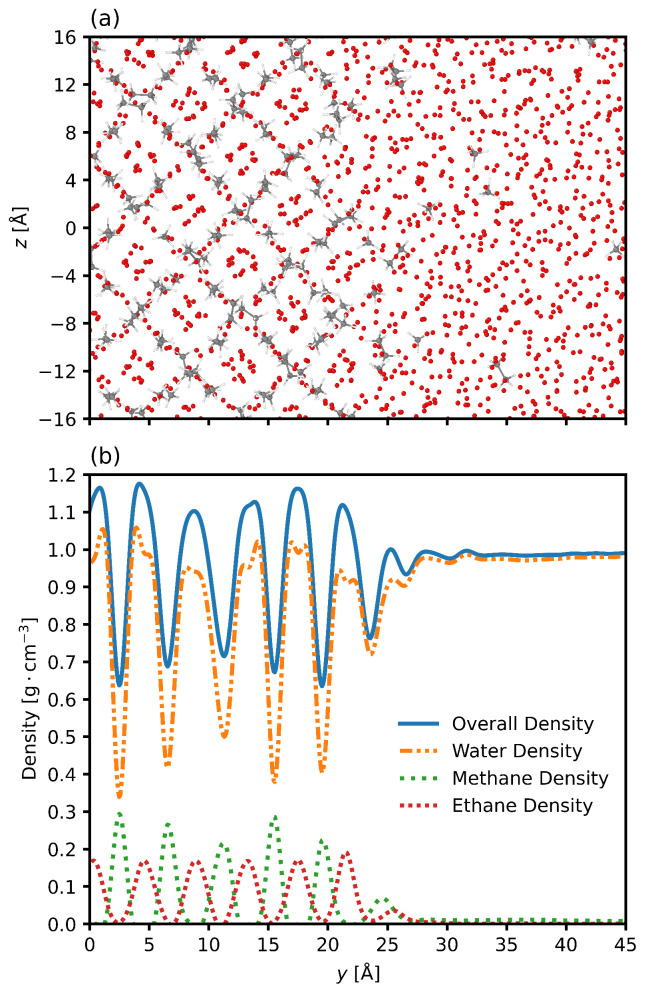
Snapshot of one interface of the system. (**a**) shows the system configuration at the final timestep. The oxygen atoms of the water molecules are visible, and the hydrogens are suppressed. Bonds of the ethane and methane molecules are shown. The system is organized into the hydrate phase around y=0 Å and transitions to bulk liquid water phase around y=20 Å. (**b**) shows the overall, water, methane, and ethane axial density distributions. The methane and ethane density peaks anterior to the solid phase indicate accumulation of the gas at the periphery of the interfacial layer. Plots are for 6.59 megapascals and 280.15 Kelvin. Samples are taken every two picoseconds. The zero point in the plot represents the center of the hydrate phase.

The density profile can provide key insight into the structure of the system and its interface, particularly in regions near the transition layer. Figure 3 shows the axial density profile of the system for one of the two interfaces. Oscillations in the hydrate phase are expected due to the periodic nature of the clathrate and are anticipated characteristics of solids made up of repeating units [81]. The density profile of the water phase is smooth and continuous, as expected for the liquid. Moving from the system center at y=0 Å to the water phase at y=45 Å, the density of the methane and ethane molecules decreases, a consequence of their low solubility in water. The transition region from solid to liquid is characterized by a decrease in the density fluctuations as the periodic nature of the hydrate is replaced by the disordered nature of the liquid.

**Figure 4:**
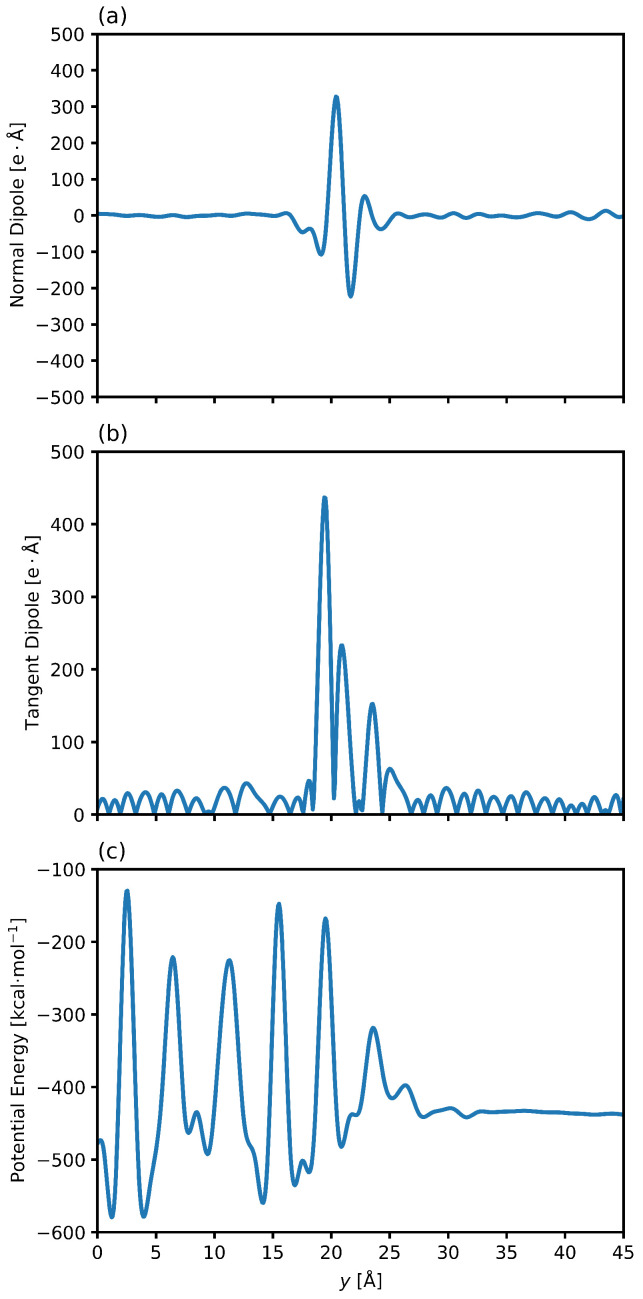
(**a**) shows the normal component of the dipole along the axis normal to the interface. There is a drop corresponding to the initial deviation from bulk hydrate, followed up oscillatory movement in the intermedial layer. (**b**) shows the tangent component of the dipole along the axis normal to the interface. Oscillations in the bulk phases correspond to instances of local organization, while the region in the center corresponds to the chaotic intermedial layer. (**c**) shows the potential energy along the axis normal to the interface. The hydrate phase shows oscillations due to the periodic and crystalline nature of the system, while the liquid water shows no net potential energy differences. The potential energy decreases across the interface. Plots are for 6.59 megapascals and 280.15 Kelvin. The system is organized into the hydrate phase around y=0 Å and transitions to bulk liquid water phase around y=20 Å. The zero point in the plot represents the center of the hydrate phase.

Many material behaviors, such as surface conductivity, chemical reactions, and adsorption, are influenced by the molecular organization at the interface. The properties of the transition layer between hydrate and liquid water depend on the balance of water-water and water-hydrate interactions [82]. With the dipole defined as a vector pointing from negative to positive, and knowing that hydrocarbons possess no net dipole [83], we seek to characterize the transitional pre-melting layer of the clathrate system by examining the spatially dependent dipole vector shown in Figure 4. The normal component reflects the degree to which the molecules align along the surface normal, while the tangential component indicates in-plane molecular ordering. Figure 4a,b show that the water molecules are predominantly disordered in the bulk water phase on the right side and go through some transition region in the center, before once again possessing no net dipole in the hydrate phase on the left side. Such behavior has been seen in previous work of hydrate/gas systems, where water molecules were found to be oriented such that the oxygen atoms were pointed towards the solid phase in some regions of the transition layer and the water was strongly parallel in other regions [49].

In this work, we see a drop in the normal component of the dipole as one moves from the solid hydrate to the liquid water phase, indicating that the water molecules are pointed with the oxygen atom towards the liquid phase, and the mirror image of this behavior is seen on the other interface. That is, the water molecules at the other interface show the same orientational behavior. However, this initial drop is then followed by a very strong orientation with the oxygen facing the other way. This behavior is also mirrored on the other interface, meaning that the water molecules are orienting themselves in alternating layers within the pre-melting layer. Importantly, this phenomenon displays the presence of an electric double layer, a feature present in ice-water systems that has not been examined for hydrate systems [84]. Figure 4b shows that the tangent component is also strongly affected by the transition across the interface. Additionally, the parallel alignment is strongest in the region closest to the solid phase while the normal component is strongest in the region closest to the liquid phase.

Such behaviors have been seen in systems of carbon nanotubes and water, where the positive charge on the surface of the tube requires that the first layer of water molecules possess an alignment with oxygen towards the solid [85]. The short distance over which these orientations fluctuate happens in water/hydrocarbon mixtures as well [86]. These behaviors were seen for all conditions simulated, with the same orientational trends. Studied for hydrocarbon systems [58], this is the first time such behaviors have been studied for water/hydrate systems in a comprehensive manner. Figure 4c shows the potential energy along the axis normal to the interface. There is a net increase in the local potential energy across the interface as we move from the liquid water phase to the crystalline hydrate phase [22]. This increase happens over a distance that is equal to the distance over which the changes in molecular dipole indicate a reorganization of the water molecules from liquid to solid.

**Figure 5:**
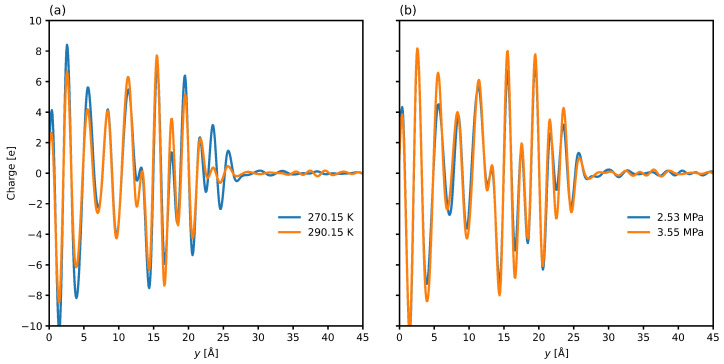
Interfacial charge distributions at different temperatures (**a**) and pressures (**b**). The interfacial layer is centered at y=23 Å with the hydrate phase on the left and the water phase on the right. The zero point in the plot represents the center of the hydrate phase.

Characterizing the interfacial polarization charge density provides another avenue to validate the orientational findings. Figure 5 demonstrates that a temperature rise or a pressure drop decreases the interface charge. This manifests due to a charge imbalance producing a negative electric field [87] and is prominent in sI hydrate/water systems as well [22]. Charge difference may impact wetting, ion transport, and adsorption, and show that the sII clathrate system is susceptible to manipulation via applying external fields. Local charge differences may also cause asymmetric molecular orientations, affecting interfacial transport properties, charge screening effects, and their relation to structuring near solid-liquid boundaries.

These novel structural findings are critical in providing a more comprehensive understanding of the molecular behaviors at the interface so that engineering applications may exploit them in relevant applications. All the aforementioned properties showed marked differences between the solid and liquid phases of the system. Characterizing the distance over which these changes occur involves finding the position and thickness of the interface, such that one can ascertain if larger scale models are capturing behaviors in the chaotic transition layer accurately.

### 3.3. Interfacial Position and Thickness

Visual detection of the interface center and thickness is not clear when simply looking at the molecular trajectory. Figure 4 shows that the normal and tangential components of the dipole vary over the transition layer, but their peak behaviors occur at slightly different positions. It is therefore useful to characterize the interfacial thickness and position, both to describe the interfacial region and ensure that the models and techniques mentioned previously are capturing the correct behaviors relative to the sharp interface. We will employ a hyperbolic tangent function to provide estimates of the interfacial thickness and position by regressing the axial density profile [35,88,89].

(5) $$\rho_{ij}\left(y\right)=\frac{1}{2}\left(\rho_{i}+\rho_{j}\right)-\frac{1}{2}\left(\rho_{i}-\rho_{j}\right)\tanh\left(\frac{|y-y_{c}|-y_{G}}{d}\right)$$

In Equation (5), *y* is the axis normal to the interface, *d* is the hlregressed measure of the thickness of the interface, $y_{c}$ is halfway between the two interfaces, $y_{G}$ is the Gibbs Dividing Surface position, and $\rho_{i}$ and $\rho_{j}$ are the bulk parameters of the two phases in question, in this case their densities. $y_{c}$ represents the center of the hydrate phase and the difference between $y_{c}$ and $y_{G}$ helps determine the position of the interface in the simulation box, and relative to other spatial phenomena to reduce numerical fluctuations in further analysis. $\rho_{i}$ and $\rho_{j}$ are regressed and compared to the actual bulk properties of the two phases to ensure that the model is capturing the correct behaviors. We can regress the thickness *d* and calculate the 10/90 thickness, which is related to the regressed thickness *d* by t=2.1972d, using Equation (5) [35]. To use Equation (5), *y* coordinates of the chunks are used as the independent variable, and the average overall density of the chunk associated to that coordinate is used as the dependent variable. $\rho_{i}$, $\rho_{j}$, $y_{c}$, and $y_{g}$ are output from the regression.

**Figure 6:**
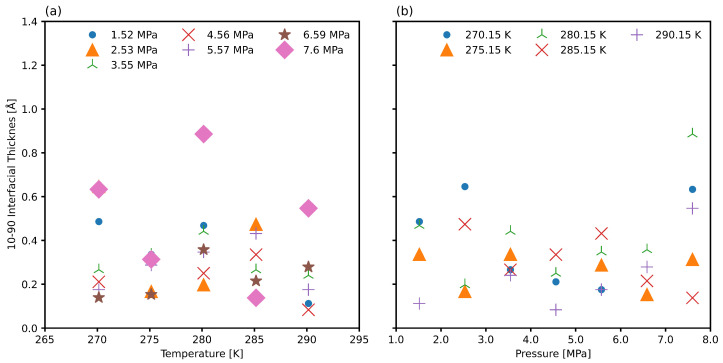
The 10/90 interfacial thickness regressed from the axial density as a function of (**a**) temperature and (**b**) pressure.

The 10/90 thickness cannot be estimated well directly for systems that have specific periodicity, as in clathrates, and therefore the regressed thickness *d* is more suitable to regress first for these systems [90]. Figure 6 presents the 10/90 thickness *t*, derived from the density regressed thickness *d*, across various temperature and pressure conditions. No significant pressure-dependent trend is observed, although the limited pressure range in this study may not fully capture variations relevant to broader engineering applications. For sII natural gas hydrate systems, interfacial thickness exhibits a weak dependence on temperature due to a combination of low simulation box expansion and the relatively small thermal expansion of hydrate crystals [48,49]. These findings indicate that the interfacial thickness for natural gas hydrate interfaces is markedly smaller than those observed in water-methane, water-ethane, and water-propane systems [29,69].

**Figure 7:**
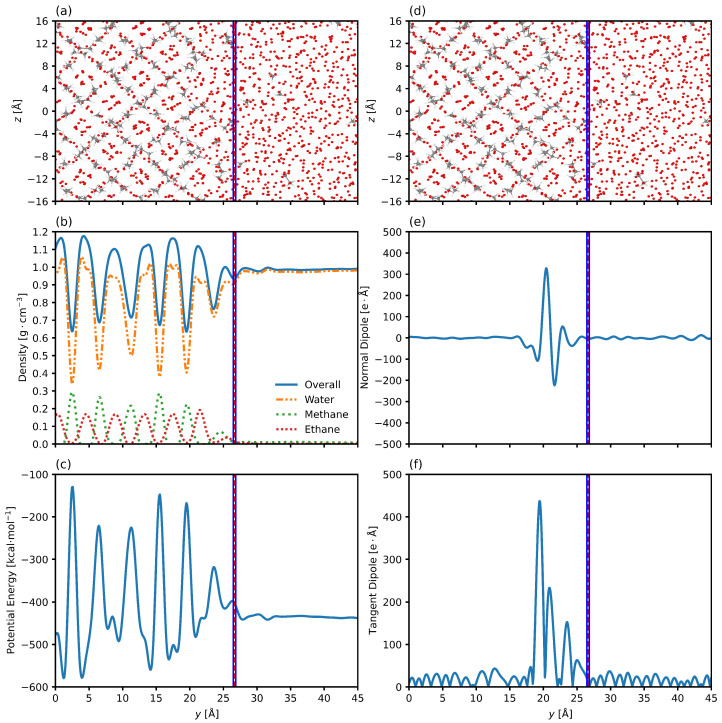
Snapshot of one interface of the system. (**a**,**d**) shows the system configuration at the final timestep with the oxygen atoms of the water molecules visible and the hydrogens suppressed, and bonds of the ethane and methane molecules are shown. (**b**) shows the axial density profile of the overall system and the three component molecules. (**c**) shows the axial potential energy profile of the system. (**e**) shows the normal dipole moment along the axis normal to the interface. (**f**) shows the tangent dipole component along the axis normal to the interface. Plots are for 6.59 megapascals and 280.15 Kelvin. The solid blue and red black lines represent the edges of the interfacial layer determined by the 10/90 interfacial thickness and the center of the interface, respectively, calculated using the regression of the axial density profile. Note that these two lines are overlapping, showing that the interfacial thickness is actually very small compared to the scale of the axis. The zero point in the plot represents the center of the hydrate phase.

The interfacial thickness regressed from the axial density profile appears relatively small when compared to estimates derived from direct visual inspection of molecular trajectories and final simulation snapshots. The discrepancy arises from the inherent limitations of the density-based approach, which relies on the spatial averaging of density profiles to extract meaningful interfacial thickness values. Initial testing of the sensitivity of the regressed thickness to the chunk size showed that there was a strong correlation between the thickness and the size when the binning thickness exceeded a few Angstroms, meaning coarse averaging causes a strong lack of numerical detail and provides misleadingly stable numbers. To rectify this, we used a chunk size of 1 Å to ensure that the thickness values were independent of the binning resolution, a key decision that is not explicitly addressed in literature studies on the topics of interfacial thickness and position. To assess the suitability of using the density regression method and the hyperbolic tangent function to estimate the thickness, we analyze how well it describes the transition in other quantities across the interface, namely the density, potential energy, normal dipole, and the tangent dipole. Figure 7 overlays the thickness and position with these quantities.

Figure 7 shows the results for the 10/90 thickness and the center of the interface as determined by the density profile regression overlaid on the system snapshot, density, potential energy, and principal dipole components. Figure 7a,d show that the interfacial center and thickness are located just at the outer edge of the quasi-liquid interfacial layer, as they display the organization of guest molecules ceases when the axial position increases beyond 26 Å. The density profile in Figure 7b shows that the interface is located just prior to becoming a liquid water phase. However, the lower amplitudes of all densities show that the regression is failing to capture the area over which these densities change, indicating that the thickness is underestimated, or at least that the center of the interface lies further towards to the hydrate phase (y<26 Å). Figure 7c shows the decrease in potential energy across the interface. The density regression once again fails to capture the full extent of the change in potential energy, indicating that the thickness is underestimated. Figure 7e,f show the normal and tangent dipole components, respectively, and clearly show that the density regression is not adequately encompassing the changes in the normal dipole, as well as the changes in the tangent dipole. The reorientation of water molecules is happening closer to the hydrate phase than the density regressed interfacial thickness and position would suggest.

Using the axial density profile as a regression target to characterize the interfacial thickness and position is a widely used approach despite possessing some shortcomings that may misrepresent the actual interfacial thickness. One primary shortcoming is the sensitivity to the chosen bin size and resolution. In MD simulations, dividing the simulation box into slabs parallel to the interface provides a way to axially average the density. Using a small slab thickness can introduce excessive noise due to local fluctuations, making it difficult to extract meaningful trends. However, a larger slab thickness obscures critical transition layers and fine structural features of the interfacial region, while also providing misleadingly stable numbers. The average diameter of cages in gas hydrates ranges from 7.82 Å for the small sII cage to 11.58 Å for the largest cage of the sH structure [91]. These should provide an absolute maximum upper bound for the slab thickness, as the interfacial layer may be composed of malformed cages or partial cages. Picking a slab thickness small enough to capture the complexities of a layer formed by these semi-cages while also being large enough to avoid excessive noise is a difficult task with no clear optimization parameter.

A second critical shortcoming of using the axial density profile is its inability to account for orientational and molecular dipole interactions. Carbon nanotubes, confined water systems, hydrocarbon/water, and other systems have shown that the interfacial layer represents both a phase transition and a region of dynamic electrostatic interactions [58,82,85,92,93]. The density may regress an artificially small interfacial thickness as it cannot represent the interfacially affected thickness—the region over which the interfacial layer affects the bulk phases. Finally, the axial density profile regression with the hyperbolic tangent struggles to differentiate interfaces where the bulk densities are similar. The average density of the sII hydrate and liquid water differ by only 0.04 $g\cdot {\mathrm{cm}}^{-3}$ [94], and therefore two of the parameters in Equation (5) are very similar and their difference becomes overcome by the short range density fluctuations that are resolved by using the small slab thickness previously mentioned. Additionally, the methane and ethane densities, while possessing a larger difference, are not as well resolved due to their low solubility in water and the small number of molecules in liquid phase.

### 3.4. Improved Measurements

**Figure 8:**
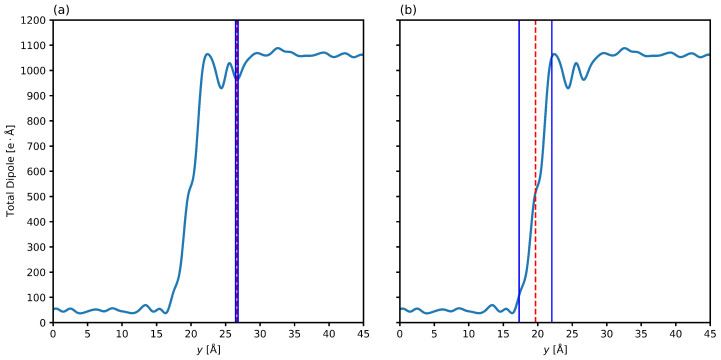
Comparison of using the axial density profile or the axial total dipole moment as a measure of interfacial location and thickness. (**a**) shows axial total dipole profile of the system, with the density regressed interfacial position and thickness overestimating the location and underestimating the thickness. (**b**) shows axial total dipole profile of the system, with the total dipole regressed interfacial position and thickness correctly estimating the position of the interface and its thickness. Plots are for 6.59 megapascals and 280.15 Kelvin. Samples are taken every two picoseconds. The zero point in the plot represents the center of the hydrate phase. The dashed red line represents the interface center and the solid blue lines encompass the 10/90 thickness.

With these limitations in mind, it becomes critical to find another way to measure the interfacial thickness and position using molecular dynamics data. The electronic structure of the water molecule yields many of its critical properties. The distribution and separation of charges in the water molecules may seem trivial but are actually exceptional and responsible for its unique capabilities. The molecule is geometrically simple and made up of oxygen and hydrate, but electronically complex [95]. The focus and importance of water’s electronic structure is therefore incongruous with the use of a simple density based measured of the interfacial thickness. Due to the proximity of the hydrate and water phase densities, the density profile fails to accurately resolve the interface. Additionally, the density profile does not take into account other charge related properties that are lauded for their responsibility in water’s properties. Therefore, we propose the employment of the hyperbolic tangent function to measure the interfacial position and thickness, but using the axial total dipole moment as the target of regression. Figure 8 shows the axial total dipole profile of the system, with the density regressed interfacial position and thickness overestimating the location and underestimating the thickness, while the total dipole regressed interfacial position and thickness (using Equation (5) with total dipole as the measurable ρ) correctly estimating the position of the interface and its thickness.

Figure 8 show that the density regressed thickness is greatly underestimated, and the position is overestimated in Figure 8a, while the total dipole regressed thickness is more accurate in Figure 8b. The latter generates a 10/90 thickness that encompasses most of the transition region from the solid hydrate phase (lower total dipole) to the liquid water phase (higher total dipole). One benefit of using the total dipole moment is that it is the vector sum of all the bond dipoles and therefore encodes some orientational information that yields a more accurate representation of the electronically affected region close to the interface. The sharp increase in structural order from the liquid water phase to the solid hydrate phase is coupled with a large decrease in rotational entropy as the molecules become restricted when they crystallize into sII hydrates [96]. Unlike the density profile, which only accounts for mass distribution, the dipole provides insight into how molecules arrange themselves even in regions with bulk densities.

This insight is critical in polar systems, such as water-hydrate interfaces, where the orientation of molecules dictates stability and structuring. The interface is not only a phase boundary but also a region of complex electrostatic interactions and the total molecular dipole will provide a more accurate representation of the interfacial thickness and position. The molecular dipole from Figure 8 also shows that the two phases have strongly different bulk values, which is a critical requirement for the hyperbolic tangent function in Equation (5) to accurately regress the interfacial thickness and position. It provides this clear signal paired with the fine axial mesh we employed, overcoming an issue of using the axial density that exists with periodic systems without smearing information by using a coarse mesh.

**Figure 9:**
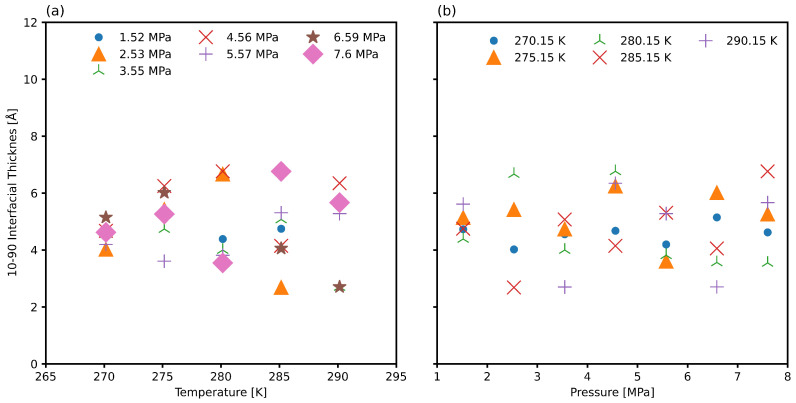
The 10/90 interfacial thickness regressed from the axial total dipole moment as a function of (**a**) temperature and (**b**) pressure.

Figure 9 presents the 10/90 thickness *t*, derived from the total dipole moment regressed thickness *d*, across various temperature and pressure conditions. As in the case of density regression from Figure 6, no significant pressure-dependent trend is observed. As expected for sII gas hydrate systems, the weak dependence on pressure is related to the low thermal expansion [48,49]. We see nearly an order of magnitude increase in the interfacial thickness compared to the density regressed thickness. This is a numerically more accurate measure as it is not dependent on the binning resolution, and it is a more physically meaningful measure as it accounts for the asymmetric distribution of molecular dipoles at the phase boundary.

**Figure 10:**
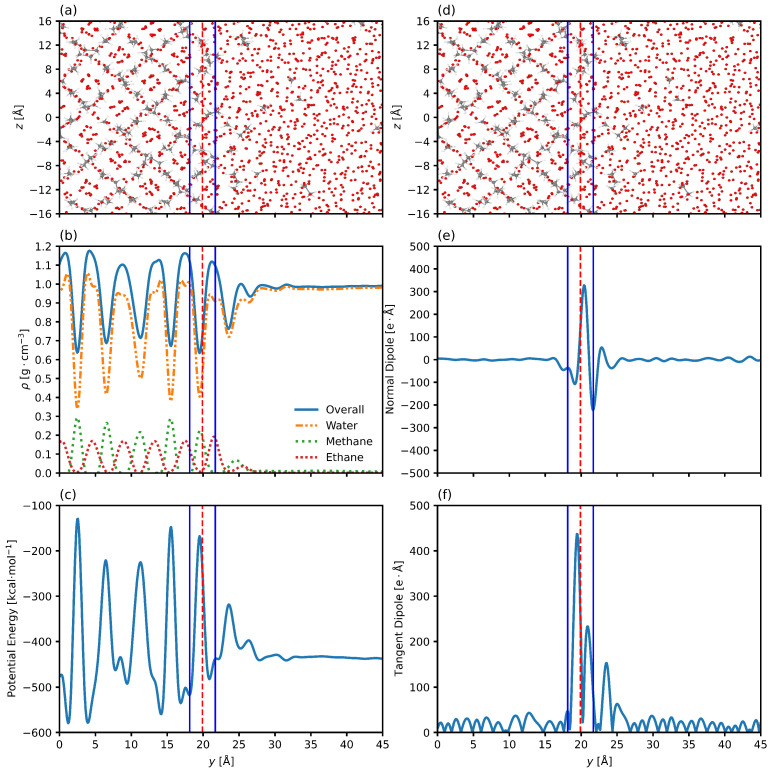
Snapshot of one interface of the system. (**a**,**d**) shows the system configuration at the final timestep with the oxygen atoms of the water molecules visible and the hydrogens suppressed, and bonds of the ethane and methane molecules are shown. (**b**) shows the axial density profile of the overall system and the three components molecules. (**c**) shows the axial potential energy profile of the system. (**e**) shows the normal dipole moment along the axis normal to the interface. (**f**) shows the tangent dipole component along the axis normal to the interface. Plots are for 6.59 megapascals and 280.15 Kelvin. The solid blue and dashed red lines represent the edges of the interfacial layer determined by the 10/90 interfacial thickness and the center of the interface, respectively, calculated using the regression of the axial total dipole moment profile. The zero point in the plot represents the center of the hydrate phase.

Data for gas hydrate interfaces is sparse in the literature. Mirzaeifard [29] reports the 10/90 thickness of water and natural gas mixtures, precursors to sII hydrate systems, to be 20 Å at 273 Kelvin to 27 Å at 303 Kelvin with a weak sensitivity to pressure. Mukherjee [96] reports that the thickness decreases with increasing density of phases when compared to the ice-water interface with the Lennard-Jones argon. Benet [97] find that the thickness of the ice-water interface is 4–5 molecular diameters (approximately 11 Å to 14 Å). Additionally, their results are presented as upper bound to results due to the small finite simulation box. Our results lie in the range of two to eight Å with a suitably sized simulation box. They are smaller than those of water/natural gas mixtures but within range of ice/water interfaces. To further display the positive qualities of employing the total dipole moment as a measure of the interfacial thickness and position, we overlay the thickness and position with the density, potential energy, and dipole components in Figure 10.

Figure 10 shows the results for the 10/90 thickness and the center of the interface as determined by the total dipole moment profile regression overlaid on the system snapshot, density, potential energy, and principal dipole components. Figure 10a,d show that the interfacial center and thickness are located at the center of the region comprised of some order due to the hydrate phase and some disorder due to the liquid phase. The density profile in Figure 10b shows that the interface is located in the center of the regions characterized by decreasing guest molecule densities. Figure 10c shows the decrease in potential energy across the interface and how the new interfacial thickness and position encompass the center of the region of change. Figure 10e,f show the normal and tangent dipole components, respectively, and clearly show that the total dipole moment regression is encompassing the changes in the normal dipole, as well as the changes in the tangent dipole. We still see some change in the dipole just outside the 10/90 interface in the liquid phase (on the right side of the dashed line), but this is likely due to the 10/90 interface not capturing the upper and lower 10% of change by definition.

Figure 10 solidifies the use of the total dipole moment as an interfacial descriptor and as a tool to ascertain both the location and thickness of an interface based on the hyperbolic tangent regression. The total dipole moment is a more physically meaningful measure of interfacial thickness as it accounts for the asymmetric distribution of molecular dipoles at the phase boundary. We generated more reliable and accurate estimates, overcoming the limitations of density-based methods and demonstrated a technique to overcome potential noise in using the density profile. The total dipole moment captures molecular orientation and electronic polarization, offering a physically meaningful representation of interfacial affected structure.

This novel approach enhances the flexibility of models across various aqueous interfaces, including water-electrolyte, water-hydrocarbon, and hydrate-forming systems by combining a fine axial mesh, a physically meaningful measure, and a regression technique that is not so sensitive to binning resolution. In complex systems where electrostatic effects are critical, like in the electric double layer that forms in many interfaces during rearrangement, using only mass distribution as an indicator yields poor results especially when the key differentiator (the densities of the two phases) are so similar, as is the case with water and hydrates.

## 4. Conclusions

We study the interfacial properties of gas hydrates using molecular dynamics simulations to provide insights required for their engineering applications and answer crucial nucleation and growth questions. The location and interface of the thickness of sII gas hydrate systems remains elusive and current methods fail to account for the complex bonding environments present in the quasi-liquid pre-melting layer, and poor measurements of these quantities lead to modeling errors and inaccurate predictions and designs. Understanding these properties is a step towards guiding engineering applications on additives and surface coverings promoting or inhibiting hydrate growth, with tunable material properties and behaviors. We compute and analyze the density profile as a function of distance to the interface, identifying heterogeneous nucleation and surface adsorption of gaseous molecules on the hydrate. We fit this data to a hyperbolic tangent function to calculate the regressed thickness and the 10/90 interfacial thickness, showing that the layer expands as temperature increases.

Since the density provides only a measure of mass and does not encode the electrostatic and orientational behaviors in the interfacial layer, it fails to account for the asymmetric distribution of molecular dipoles at the phase boundary. We then used the total dipole moment as a target of the regression, showing that it provides a significantly more accurate representation of the interfacial thickness and position due to its more physically meaningful nature and accurate measurements. The total dipole moment captures molecular orientation and electronic polarization, offering a physically meaningful representation of interfacially affected structure. This new approach enhances the flexibility of models across various aqueous interfaces, including water-electrolyte, water-hydrocarbon, and hydrate-forming systems by combining a fine axial mesh, a physically meaningful measure, and a regression technique that is not so sensitive to binning resolution. Future work is required to evaluate the effect of the imposition of system behaviors that comes with specifying a regression function, as well as utilizing different observables that would be suitable in systems without such differences in the total dipole moment.

## Figures and Tables

**Table 1:** Force field parameters for methane, ethane, and water molecules.

Molecule	Quantity [Units]	Value
CH_4_/C_2_H_6_	C mass [g·mol^−1^]	12.011
	H mass [g·mol^−1^]	1.008
	CC ϵ [kcal·mol^−1^]	0.066
	CH ϵ [kcal·mol^−1^]	0.03
	CC σ [Å]	3.5
	CH σ [Å]	2.5
H_2_O	O mass [g·mol^−1^]	15.999
	H mass [g·mol^−1^]	1.008
	O charge [e]	−1.04844
	H charge [e]	0.52422
	OO ϵ [kcal·mol^−1^]	0.16275
	OH, HH ϵ [kcal·mol^−1^]	0.0
	OO σ [Å]	3.16435
	OH, HH σ [Å]	1.0
	OH r_0_ [Å]	0.9572
	HOH *θ*_0_ [°]	104.52

## Data Availability

Data is contained within the article.
